# Renal arteriovenous malformation mimicking hydronephrosis—hidden danger

**DOI:** 10.1259/bjrcr.20190034

**Published:** 2019-11-15

**Authors:** Chung Shen Chean, Jia Ying Kuah, Martin Stopa, John Asquith, Anurag Golash, Cherian George

**Affiliations:** 1 Imaging Department, Royal Stoke University Hospital, Newcastle Road, Stoke-on-Trent ST4 6QG, United Kingdom; 2 Urology Department, Royal Stoke University Hospital, Newcastle Road, Stoke-on-Trent ST4 6QG, United Kingdom

## Abstract

Renal arteriovenous malformations (AVMs) are rare, with an incidence of approximately 0.04%. Diagnosis is often challenging due to mimics of AVMs. We report a case of renal AVM mimicking hydronephrosis on ultrasound and unenhanced computed tomography (CT). A 24-year-old female with background of recurrent urinary tract infections (UTIs) presented to the Accident and Emergency department with 1 day history of bilateral flank pain, dysuria, rigors and pyrexia. Urine dipstick showed microscopic haematuria and blood tests showed mild neutrophilia. Dilated right renal pelvis was seen on ultrasound. Unenhanced CT of the urinary tract demonstrated right hydronephrosis with no evidence of calculi. Subsequent Uro-radiology meeting discussion concluded that renal pelvis might be pus-filled and recommended an urgent nephrostomy. However, ultrasound Doppler scan performed at the time of the planned nephrostomy demonstrated colour flow within dilated renal pelvis suggestive of an AVM. Nephrostomy was abandoned and subsequent CT angiogram confirmed a large congenital AVM. The patient was referred for embolization.Colour flow ultrasound imaging is a simple and quick technique to diagnose AVMs. However, as in our case, when colour flow Doppler imaging was not used at the initial ultrasound, the opportunity to obtain an accurate diagnosis was missed. If the subsequently planned nephrostomy had taken place, this may have led to potentially serious outcomes. We suggest that colour flow imaging should be used prior to nephrostomy insertion to differentiate hydronephrosis from vascular abnormalities.

Renal arteriovenous malformation (AVM) is an abnormal communication between arteries and veins lying underneath urothelium with the absence of capillary bed. Renal AVM has a prevalence of 0.04% and it usually presents in late adult life.^1^ It has varied presentation, thus causing difficulty to establish a radiographic diagnosis. We describe a case of renal AVM mimicking hydronephrosis on ultrasoundand CT of kidneys, ureters and bladder (CT-KUB) in a 24 year old female with recurrent urinary tract infections (UTI).

## Clinical presentation

A 24-year-old female presented to the accident and emergency (A&E) department with bilateral flank pain, dysuria, rigors and fever of 24 h duration. She was normally fit and well with no past medical history, other than recurrent UTI. She was on no medication and had no history of surgery or trauma. On physical examination, her abdomen was soft with mild tenderness in the flanks bilaterally. No abdominal masses were palpable and there were no audible bruit. Her temperature at presentation was 38.8°C. On admission, she was found to have microscopic haematuria on urine dipstick and her blood results showed only mild neutrophilia and normal estimated glomerular filtration rate (eGFR). The rest of the examination was unremarkable.

**Figure 1.**
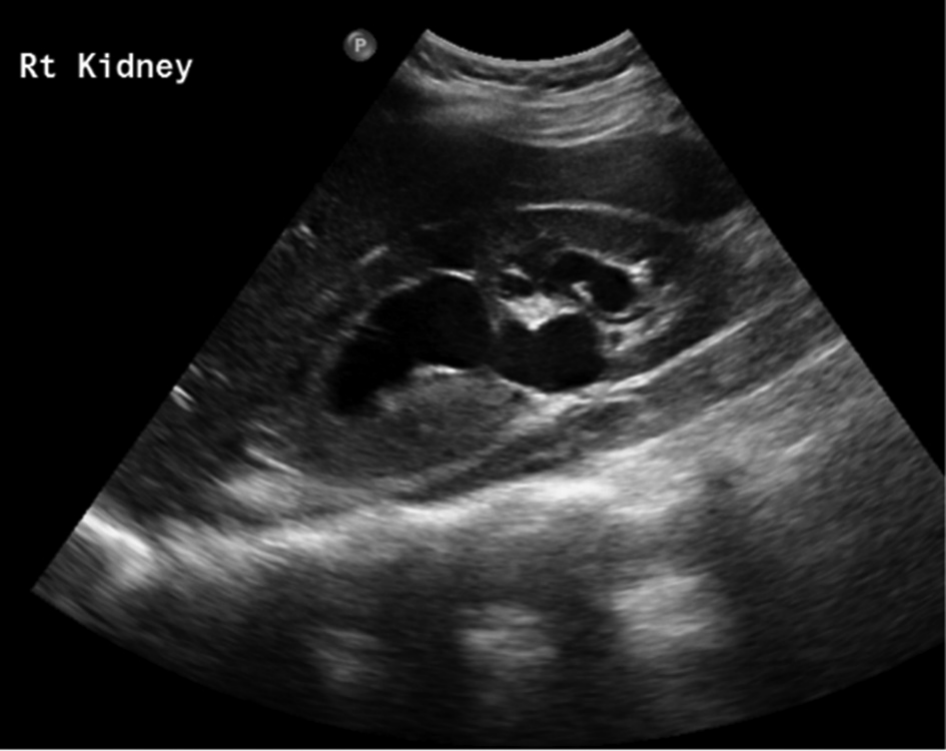
Ultrasound-KUB showing moderate to severe right hydronephrosis with no evidence of renal calculi. Ultrasound KUB,Ultrasound of kidneys, ureters and bladder.

A recent outpatient ultrasound scan performed on this patient showed a dilated right renal pelvis, with right kidney measuring 13.7 cm and left kidney measuring 11.5 cm with no other abnormality (Figure 1).

## Differential diagnosis, Investigations and Management

**Figure 2.**
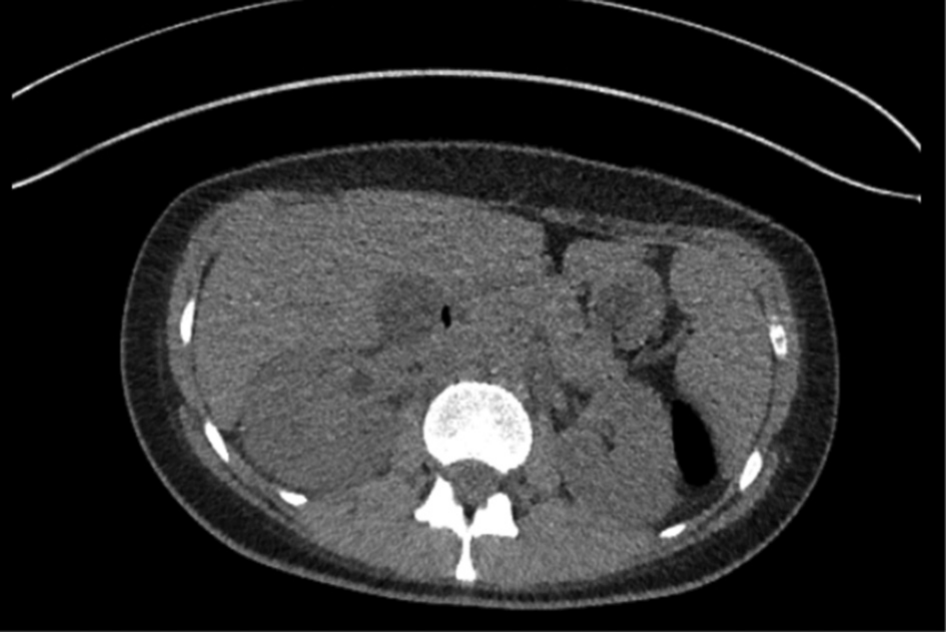
Axial CT-KUB with no evidence of renal calculi but hydronephrosis with suggestion of a pus-filled right renal pelvicalcyeal system. CT-KUB,CT of kidneys, ureters and bladder.

Given the history and presentation, a CT-KUB was performed for suspected obstructing urinary tract calculi. The CT scan demonstrated right hydronephrosis but no evidence of calculi or other causes of obstruction, although sepsis related pelvic–-ureteric junction obstruction due to debris was postulated (Figure 2).

The case was discussed the next day at the urology–radiology multidisciplinary team (MDT) meeting and it was thought that possible differentials for the appearances of the right kidney from the non-contrast imaging include pyonephrosis or a passed calculus. The differential of parapelvic cysts mimicking hydronephrosis was also considered given its appearances on non-contrast imaging. However, from the patient’s initial clinical presentation, the diagnosis of parapelvic cysts was thought to be less likely. An urgent nephrostomy was thought to be the best option to drain the suspected pyonephrosis.

**Figure 3.**
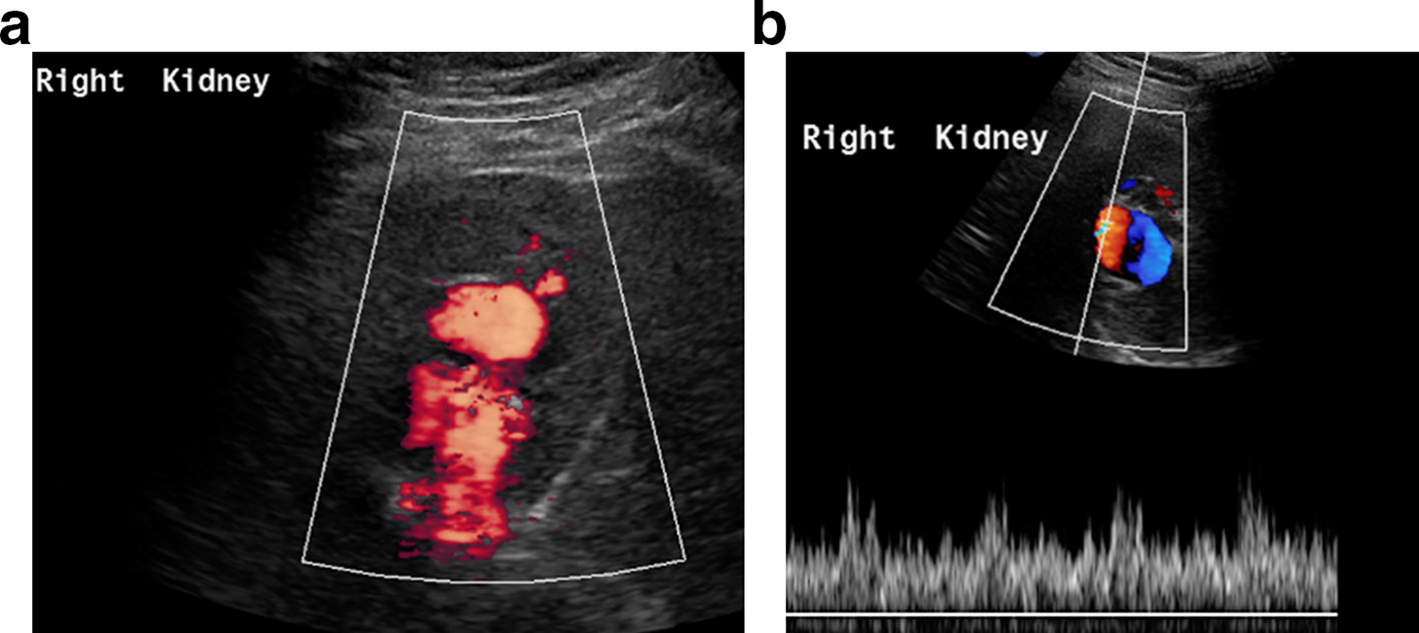
(a, b) A large AVM in the right kidney demonstrated by ultrasound colour flow imaging. AVM, arteriovenous malformation.

When the patient was brought down to the interventional suite for the nephrostomy, an ultrasound Doppler scan was performed by the radiologist to plan the nephrostomy access. This demonstrated significant colour flow and mixture of arterial and venous flow within the presumed dilated renal pelvis with a feeding vessel suggestive of a large AVM (Figure 3).

**Figure 4.**
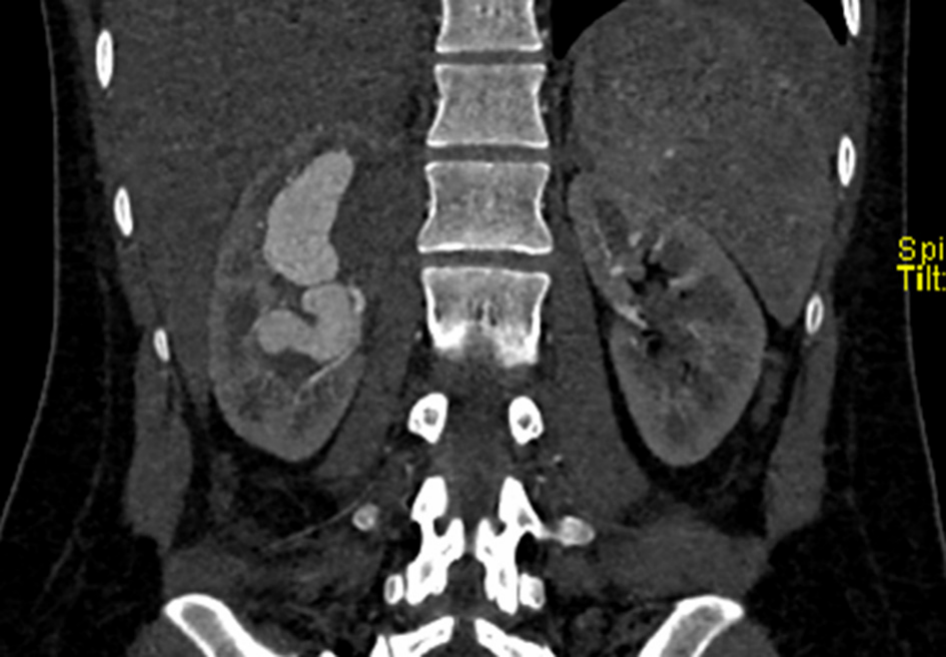
CT angiogram of the kidneys: large lobulated vascular mass within the right kidney. Suspicion of arterial feeding vessel and a draining vein at lower pole, suggestive of an AVM. Right upper pole focal pyelonephritis with reactive right pleural effusion were also evident. AVM, arteriovenous malformation.

The nephrostomy was abandoned and a CT angiogram of the kidneys and abdomen was performed which confirmed a congenital AVM, with evidence of focal pyelonephritis involving the medial and anteromedial upper pole of the right kidney. There was no evidence of hydronephrosis (Figure 4).

### Outcome and follow-up

The patient was better after 3 days of treatment with antibiotics and was discharged home with a referral to a more experienced regional centre for consideration for embolisation.

## Discussion

Renal AVMs are rare entities and have low prevalence. Despite its relatively low-reported prevalence, actual prevalence might be higher as many patients have no symptoms of the disease.^2^ It may be classified into congenital or acquired. Congenital renal AVMs account for up to 27% of all AVMs and are classed into cirsoid (multibranched) and aneurysmal configuration that is defined by the fistulas appearing as solitary communications between the artery and vein, with cirsoid morphology predominating 3:1.^3,4^ These lesions are almost always unilateral, predominantly in the right kidney.^5^ They are usually asymptomatic until the third or fourth decade and the most common presentation is with haematuria, flank or back pain and hypertension.^3,6,7^

AVMs have been shown to mimic a variety of renal pathologies including hydronephrosis, renal tumours and cysts.^6,8,9^ Often the initial diagnosis is missed, and the AVM is picked up later in the course of the patients’ investigative pathway. This can sometimes lead to unnecessary investigations or potentially have serious consequences if the diagnosis is not made correctly.

Our patient suffered with recurrent UTI and presented with microscopic haematuria and flank pain on this admission mimicking renal calculi. However, the CT-KUB did not demonstrate any calculi but only right hydronephrosis. As she had pyrexia and signs of infection, pyelonephritis with pus filled collecting systems was the primary diagnosis, although differential diagnosis of parapelvic cysts mimicking hydronephrosis was also considered. It was not until the ultrasound colour Doppler that was used at the planned nephrostomy insertion that significant colour flow was demonstrated and an AVM was diagnosed. We routinely use colour flow imaging prior to any ultrasound-guided biopsies or drainages to map out intralesional or perilesional vascularity thereby helping to avoid potential procedure-related haemorrhage.

Colour flow ultrasound Doppler imaging has been shown in the literature to be highly effective at diagnosing AV malformations and is considered to be the first imaging study useful in patients with or suspected of having AVM. It has the advantage of being low cost and less invasive.^10,11^ However, on grey scale ultrasound imaging, the hydronephrosis may mimic a variety of pathologies.^8,12,13^

Colour flow Doppler imaging was not used at the time of the initial ultrasound and the lack of contrast in the CT-KUB led to the incorrect diagnosis of hydronephrosis. If the planned nephrostomy had been done, the patient may have had a potentially life-threatening bleeding, considering that the AVM would have been punctured. The use of colour flow imaging at the time of the nephrostomy helped to avert a potentially serious complication.^10^

## Learning points

- Renal AVM may mimic other pathologies and a high index of suspicion is required.
- Colour flow imaging is simple and quick technique that is proven to be effective at detecting AVMs.
- If used at the time of scanning before the insertion of a nephrostomy, vascular abnormalities may be differentiated from hydronephrosis, thereby avoiding potentially life-threatening complications.

## Consent

Written informed consent was obtained from the patient for publication of this case report, including accompanying images. A copy of the written consent from patient is available, on request by the journal.
